# A new method for maturity-dependent fractionation of neutrophil progenitors applicable for the study of myelodysplastic syndromes

**DOI:** 10.1186/2050-7771-2-2

**Published:** 2014-01-22

**Authors:** Huiyuan Hu, Yayoi Shikama, Tsutomu Shichishima, Kazuhiko Ikeda, Kazuko Akutsu, Tomoyuki Ono, Hideo Kimura, Kazuei Ogawa, Hideyoshi Noji, Yasuchika Takeishi, Junko Kimura

**Affiliations:** 1 Department of Pharmacology, Fukushima Medical University School of Medicine, 1 Hikarigaoka, Fukushima 960-1295, Japan; 2 Department of Pharmaceutical Toxicology, China Medical University School of Pharmaceutical Sciences, Shenyang, China; 3 Department of Cardiology and Hematology, Fukushima Medical University, Fukushima, Japan; 4 Fukushima Research Institute of Environment and Medicine, Futaba, Japan; 5 Department of Hematology, Iwaki Kyoritsu General Hospital, Iwaki, Japan; 6 Department of Hematology, Kita Fukushima Medical Center, Date, Japan

**Keywords:** Myelodysplastic syndromes, AML1, C/EBP-ϵ, Granulopoiesis, Fractionation, Gene expression profile

## Abstract

We applied our new method, maturity-dependent fractionation of bone marrow-derived neutrophil progenitors, to a study of gene expression profiles during granulopoiesis in myelodysplastic syndromes. CD34^+^ cells with low density [F1], CD11b^-^/CD16^-^ [F2], CD11b^+^/CD16^-^ [F3] and CD11b^+^/CD16^low^ [F4] with intermediate density, CD11b^+^/CD16^int^ [F5] and CD11b^+^/CD16^high^ [F6] with high density were isolated from six patients. Although AML1 and C/EBP-ϵ mRNA peaked at F1 and F4, respectively, in healthy individuals, C/EBP-ϵ was maximized at F2/F3 in all patients, two of whom showed simultaneous peaks of AML1 at F2. Thus, this fractionation is useful to detect mistimed induction of granulopoiesis-regulating genes in myelodysplastic syndromes.

## To the editor

Myelodysplastic syndromes (MDS) are clonal disorders of hematopoietic stem cells characterized by cytopenia with dysplastic phenotypes of myeloid cells [1], which have been thought to result from defective differentiation [2]. However, the molecular basis of impaired differentiation is largely unclear. Since *in vivo* gene expression profiles during granulopoiesis in MDS have not been studied because of the lack of an appropriate method to fractionate progenitors, we applied our newly established method that separates bone marrow (BM)-derived neutrophil progenitors into six sequential maturation stages [3] for BM specimens of six low-risk MDS patients (Table 1). All volunteers provided a written form of informed consent in accordance with the institutional Human Research Committee and Helsinki Declaration.

**Table 1:** Clinical and hematological findings and treatment in six patients with MDS

**Diagnosis**	**Age/sex**	**WBC (×10**^**9**^**/L)**	**ANC (×10**^**9**^**/L)**	**Hb (g/dL)**	**PLT (×10**^**9**^**/L)**	**BM NCC (×10**^**9**^**/L)**	**Karyotypes**	**Therapy**
RCUD	74/M	4.5	3.5	8.4	206	143	46,XY	None
RCMD 1	66/M	2.4	1.7	9.2	46	37	46,XY	None
RCMD 2	79/F	3.5	2.0	10.3	6	50	46,XX	RBC and platelet transfusion
RCMD 3	73/M	2.8	1.4	10.3	79	207	46,XY,del(20)(q11)	None
RCMD 4	26/F	4.0	0.9	11.2	50	35	46,XX	None
RARS	83/M	4.8	3.4	6.1	336	240	46,XY	None

*Abbreviations*; *WBC* white blood cell counts, *ANC* absolute neutrophil counts, *Hb* concentration of hemoglobin, *PLT* platelet counts.

*BM NCC* bone marrow nucleated cell counts, *M* male, *F* female, *RBC* red blood cells, *RCUD* refractory cytopenia with unilineage dysplasia, *RCMD* refractory cytopenia with multilineage dysplasia, *RARS* refractory anemia with ringed sideroblasts.

Heparinized BM blood was divided into low (<1.065 g/mL), intermediate (between 1.065 g/mL and 1.080 g/mL), and high density (>1.080 g/mL) populations. The overall recovery rate (73.7 ± 12.4%) did not differ from the results of healthy BM [3]. Non-neutrophil lineages expressing CD2, CD3, CD14, CD19, CD56, CD61, glycophorin-A, or CD49d were eliminated, and the remaining cells were subjected to immunostaining for fluorescence activated cell sorting of CD34^+^ cells with low density [F1], CD11b^-^/CD16^-^ [F2], CD11b^+^/CD16^-^ [F3], and CD11b^+^/CD16^low^ [F4] with intermediate density, as well as CD11b^+^/CD16^int^ [F5] and CD11b^+^/CD16^high^ [F6] with high density. The six fractions derived from MDS patients well correlated with morphological maturation of neutrophil progenitors (Table 2), as previously shown using healthy BM.

**Table 2:** Morphological counts of the cells included in sorted fractions

	**MB**	**PM**	**MC**	**MM**	**SB**	**SE**	**Others**
F1	69.7 ± 23.7%	29.3 ± 22.8%	0.3 ± 0.3%	0.0 ± 0.0%	0.0 ± 0.0%	0.0 ± 0.0%	0.7 ± 0.6%
F2	0.0 ± 0.0%	47.8 ± 16.1%	55.8 ± 17.1%	0.5 ± 0.4%	0.3 ± 0.4%	0.0 ± 0.0%	2.5 ± 4.8%
F3	0.2 ± 0.4%	0.5 ±0.5%	80.1 ± 18.0%	11.4 ± 14.7%	1.3 ± 1.9%	0.2 ± 0.5%	5.7 ± 11.4%
F4	0.0 ± 0.0%	0.3 ± 0.8%	70.3 ± 29.0%	24.6 ± 20.6%	4.3 ±5.9%	0.4 ± 0.7%	0.0 ± 0.0%
F5	0.0 ± 0.0%	0.0 ± 0.0%	17.4 ± 8.3%	29.8 ± 20.1%	49.4 ± 24.3%	7.9 ± 7.2%	1.3 ± 3.0%
F6	0.0 ± 0.0%	0.0 ± 0.0%	1.1 ± 1.5%	7.0 ± 4.9%	57.5 ± 11.5%	34.4 ± 15.7%	0.1 ± 0.2%

The numbers represent mean ± SD. MB; myeloblasts, PM; promyelocytes, MC; myelocytes, MM; metamyelocytes, SB; neutrophils with stab nuclei, SE; neutrophils with segmented nuclei, others: non-neutrophilic lineages including erythroblasts, monocytes, lymphocytes, and eosinophils.

**Figure 1:**
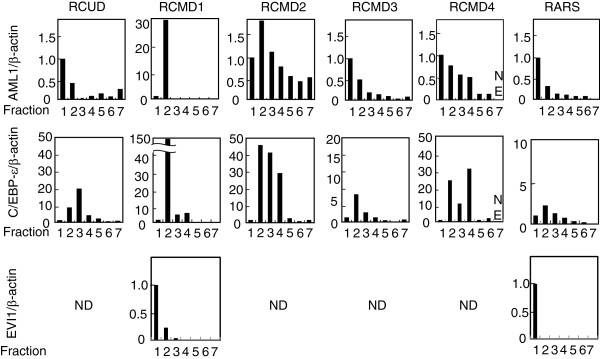
**Expression profiles of AML1, C/EBP-ϵ, and EVI1 mRNA in each MDS patient.** The ratio of AML1, C/EBP-ϵ, or EVI1 to β-actin mRNA in F1 was plotted as 1.0 in each panel. NE; not examined, ND; not detected.

The BM-derived fractions and a peripheral blood-derived neutrophil fraction [F7] were subjected to quantification of mRNA of acute myeloid leukemia 1 (AML1), CCAAT-enhancer-binding protein-epsilon (C/EBP-ϵ), and ecotropic viral integration site 1 (EVI1) as previously described [3]. As shown in Figure 1, four out of the six patients showed peak expressions of AML1 mRNA at F1 followed by a gradual decrease as previously shown in healthy BM [3]. However, two patients, refractory cytopenia with multilineage dysplasia (RCMD)1 and RCMD2, showed maximum AML1 expressions at F2, which were 28- and 1.8-fold higher than those at F1, respectively. The delayed peak of AML1 mRNA was not due to the contamination of myeloblasts (MBs) in F2, since RCMD1- and RCMD2-derived F2 consisted of mostly promyelocytes (PMs) and myelocytes (MCs), with no MBs. The peak expression of C/EBP-ϵ mRNA was detected at F2 in four patients and at F3 in one patient, while the other patient showed two peaks at F2 and F4. In healthy BM, C/EBP-ϵ mRNA was maximally expressed at F4 [3] following the acquisition of cell-surface expression of CD11b. Simultaneous peaks of C/EBP-ϵ and AML1 at F2 in RCMD1 and RCMD2 were obviously abnormal. The remarkable dropdown and absence of EVI1 mRNA at F2 of RCMD1 and RCMD2, respectively, suggested that both AML1 and C/EBP-ϵ mRNA were specifically increased at F2 in these patients.

These aberrant gene inductions were not detectable by analyses of only a hematopoietic stem cell fraction [4,5], or density-based three fractions [6] which includes F2, F3, and F4 in the same fraction with intermediate density. Thus, our newly established method of neutrophil progenitor fractionation is applicable to MDS, and may provide a new insight into understanding the molecular basis of impaired differentiation.

## Competing interests

There are no relevant conflicts of interest to disclose for any of the authors.

## Authors’ contributions

H H performed this research. Y S designed this study, analyzed the data, and wrote the paper. T S collected patients’ samples and revised the paper critically. K I and K A performed cell isolation by fluorescence-activated cell sorter. T O took part of density centrifugation and immunostaining. H K, K O, and H N chose appropriate patients and collected the specimens. Y T and J K critically discussed this study and revised the paper. All authors read and approved the final manuscript.
